# Variability of [^18^F]FDG-PET/LDCT reporting in vascular graft and endograft infection

**DOI:** 10.1007/s00259-023-06349-3

**Published:** 2023-07-29

**Authors:** David J. Liesker, Stijn Legtenberg, Paola A. Erba, Andor W. J. M. Glaudemans, Clark J. Zeebregts, Jean-Paul P. M. De Vries, Nabil Chakfé, Ben R. Saleem, Riemer H. J. A. Slart

**Affiliations:** 1 Department of Surgery (Division of Vascular Surgery), University Medical Center Groningen, University of Groningen, PO Box 30.001, 9700 RB Groningen, The Netherlands; 2 Nuclear Medicine and Molecular Imaging, University Medical Center Groningen, University of Groningen, PO Box 30.001, 9700 RB Groningen, The Netherlands; 3 Department of Nuclear Medicine, Papa Giovanni XXIII Hospital, Bergamo, Italy; 4 Department of Vascular Surgery and Kidney Transplantation, Hôpitaux Universitaires de Strasbourg, Université de Strasbourg, Strasbourg, France; 5 Department of Biomedical Photonic Imaging, University of Twente, Enschede, The Netherlands

**Keywords:** [^18^F]FDG-PET/LDCT, Vascular graft and endograft infection, Reporting, Diagnostic imaging

## Abstract

**Purpose:**

^18^F-fluoro-D-deoxyglucose positron emission tomography with low dose and/or contrast enhanced computed tomography ([^18^F]FDG-PET/CT) scan reveals high sensitivity for the diagnosis of vascular graft and endograft infection (VGEI), but lower specificity. Reporting [^18^F]FDG-PET/CT scans of suspected VGEI is challenging, reader dependent, and reporting standards are lacking. The aim of this study was to evaluate variability of [^18^F]FDG-PET/low dose CT (LDCT) reporting of suspected VGEI using a proposed standard reporting format.

**Methods:**

A retrospective cohort study was conducted including all patients with a suspected VGEI (according to the MAGIC criteria) without need for urgent surgical treatment who underwent an additional [^18^F]FDG-PET/LDCT scan between 2006 and 2022 at a tertiary referral centre. All [^18^F]FDG-PET/LDCT reports were scored following pre-selected criteria that were formulated based on literature and experts in the field. The aim was to investigate the completeness of [^18^F]FDG-PET/LDCT reports for diagnosing VGEI (proven according to the MAGIC criteria) and to evaluate if incompleteness of reports influenced the diagnostic accuracy.

**Results:**

Hundred-fifty-two patients were included. Median diagnostic interval from the index vascular surgical procedure until [^18^F]FDG-PET/LDCT scan was 35.5 (7.3–73.3) months. Grafts were in 65.1% located centrally and 34.9% peripherally. Based on the pre-selected reporting criteria, 45.7% of the reports included all items. The least frequently assessed criterion was FDG-uptake pattern (40.6%). Overall, [^18^F]FDG-PET/LDCT showed a sensitivity of 91%, a specificity of 72%, and an accuracy of 88% when compared to the gold standard (diagnosed VGEI). Lower sensitivity and specificity in reports including ≤ 8 criteria compared to completely evaluated reports were found (83% and 50% vs. 92% and 77%, respectively).

**Conclusion:**

Less than half of the [^18^F]FDG-PET/LDCT reports of suspected VGEI met all pre-selected criteria. Incompleteness of reports led to lower sensitivity and specificity. Implementing a recommendation with specific criteria for VGEI reporting is needed in the VGEI-guideline update. This study provides a first recommendation for a concise and complete [^18^F]FDG-PET/LDCT report in patients with suspected VGEI.

## Introduction

Vascular graft and endograft infection (VGEI) is a major complication of vascular surgery and is associated with high morbidity and mortality [1, 2]. The incidence of VGEI is difficult to assess, because the aetiology of this complication is complex and multifactorial including patient-related risk factors and pre-, intra-, and post-operative factors [3]. To improve early diagnosis and clinical outcomes, adequate treatment is important. However, diagnosis can be complicated due to the inability to take microbiological cultures because of a complex anatomical location or due to a subtle and non-specific clinical presentation [4, 5]. VGEI can present early (within 4 months) or late (> 4 months). Especially late VGEI can be challenging to diagnose due to lack of systemic signs of infection or elevated white blood cell count [6].

In patients with a suspected VGEI, computed tomography angiography (CTA) is usually the preferred imaging modality to be performed [6]. However, nuclear medicine modalities may be needed to confirm the diagnosis and to analyse the extent and possible spread of the infection [7]. Literature has shown that ^18^F-fluoro-D-deoxyglucose positron emission tomography with low-dose and/or contrast-enhanced computed tomography ([^18^F]FDG-PET/CT) scan reveals a high sensitivity for the diagnosis of VGEI, but it should be performed preferably at least 4 months post-operative to avoid false positive findings [3, 5, 7]. False positive results can be caused by physiologic FDG uptake due to a sterile inflammatory response after surgery [5, 7].

Reporting [^18^F]FDG-PET/CT scans of suspected VGEI is challenging, reader dependent, and report standards or interpretation criteria are still lacking. In contrast to VGEI, reporting standards on other specialities (e.g. oncology) are already available for a decade, are widely used, and are known to improve clinical outcomes [8–10]. Interpretation of [^18^F]FDG-PET/CT scans in VGEI patients can be performed in many ways: (1) visually, by uptake pattern (focal vs diffuse), uptake intensity, uptake outside vessel boundaries, uptake in regional lymph nodes; and/or (2) semi-quantitatively by SUV measurements, by comparison (ratios) with for example blood pool or liver. Different interpretation criteria exist, but no standardisation of these criteria is accepted yet [11, 12]. Therefore, the aim of this study was to evaluate variability of [^18^F]FDG-PET/low dose CT (LDCT) reporting of suspected VGEI using a proposed standard reporting format based on findings in current literature and to evaluate if incompleteness of reports influenced the diagnostic accuracy.

## Material and methods

### Subjects

All consecutive patients with a suspected VGEI who underwent a [^18^F]FDG-PET/LDCT-scan, at the University Medical Centre Groningen (UMCG) between September 2006 and September 2022, were included. Patients with an age below 18 years old were excluded.

Suspicion of VGEI was defined as undefined fever, localised clinical features of graft infection (e.g. erythema, swelling, warmth, pain, and purulent discharge), elevated infectious variables in laboratory analysis (erythrocyte sedimentation rate, CRP, and white blood cell count), undefined malaises, positive blood cultures, and positive microbiology cultures in patients with previously implanted prosthetic grafts, as defined by the Management of Aortic Graft Infection (MAGIC) criteria [13]. Diagnosed VGEI according to the MAGIC criteria (VGEI was proven if there was at least one single major criterion and any other criterion from another category) was the gold standard.

The institutional review board approved dispensation in accordance with Dutch law on patient-based medical research obligations (registration no. METc 2022/453). Consequently, informed consent was not obtained. All patient-related data were processed anonymously and stored electronically in agreement with the Declaration of Helsinki—Ethical principles for medical research involving human subjects [14].

### Data extraction

Data were extracted from the electronic patient files (Epic Hyperspace®, Epic Systems Corporation). Suspected VGEI patients were identified through searches on intervention codes and International Statistical Classification of Diseases and Related Health Problems (ICD-10) codes.

### Patient characteristics

Baseline patient characteristics included age (years) at time of [^18^F]FDG-PET/LDCT, sex, body mass index (BMI), tobacco use, hypertension, hyperlipidaemia, and diabetes mellitus. The comorbidities were classified by the Society for Vascular Surgery (SVS) system (classes 0–3, for grading factor severity from absent to severe) according to the Ad Hoc Committee on Reporting Standards and were scored positive if the status was ≥ 1 [15].

### Surgical procedure and [^18^F]FDG-PET/LDCT scan

Vascular graft location was divided into central (aortoiliac position) or peripheral (other positions) and the surgical procedure into open repair and endovascular repair. The interval (months) between the index surgical procedure and the first [^18^F]FDG-PET/LDCT in case of a suspected VGEI was calculated. Based on this interval the scan was labelled as early (≤ 4 months post-operative) or late (> 4 months post-operative). Furthermore, the use of antibiotics at time of [^18^F]FDG-PET/LDCT was noted.

### VGEI treatment

The interval between the first [^18^F]FDG-PET/LDCT scan and any surgical VGEI treatment (e.g. graft replacement) was calculated.

### [^18^F]FDG-PET-acquisition, image analysis, and reporting assessment

[^18^F]FDG-PET-scan imaging, whole body mode (i.e. from either the sole or halfway up the thigh to the crown of the head) was performed on two different PET/CT scanners (Biograph Vision or mCT40, Siemens Healthineers, Erlangen, Germany). All scans were performed and reconstructed according to EANM guidelines [9]. Patients received FDG intravenously based on their weight (3 MBq kg − 1), while fasted for at least 6 h prior to scanning. All scans were performed 60 min after injection of ^18^F-FDG. An additional continuous breathing low-dose CT (80–120 kV, 20–35 mAs, and 5 mm slice thickness) was performed for attenuation correction and visualisation of anatomical structures. Data was processed using standard software, applying an iterative reconstruction algorithm. For patients that received multiple [^18^F]FDG-PET/LDCT scans during the diagnostic process, the first one was used as a baseline. The first scan was used to keep the influence of antibiotic treatment as small as possible and to create a homogenous cohort. All [^18^F]FDG-PET/LDCT images have been analysed by a nuclear medicine physician. The original reports of the [^18^F]FDG-PET/LDCT scans were used. The reporting nuclear physicians were noted and the years of experience during reporting were calculated.

All original [^18^F]FDG-PET/LDCT reports were scored following pre-selected (by authors BS and RS) criteria (Table 1) that were formulated based on literature [7–9, 12]. General criteria were based on the reporting guidance for [^18^F]FDG-PET/CT imaging in oncology and included comparison to other diagnostic imaging modalities (if available), area of interest (i.e. total or part of the prosthesis involved, specific part described), uptake intensity (i.e. 1. uptake similar to the background; 2. low uptake, comparable with inactive muscles and fat; 3. moderate uptake, higher than the uptake in group 2, but distinctly less than physiologic uptake by the bladder; 4. strong uptake, comparable to the uptake in the bladder), demarcation (i.e. which vessel, what side), comparison to physiological distribution (i.e. liver, spleen, digestive tract, ureters, and bladder), and body compartments [11]. Specific criteria for VGEI-related complications regarding inflammation and infection included locoregional involvement (e.g. lymph nodes, abscess, soft tissue induration), organ involvement (e.g. enteric fistula), prosthesis involvement (i.e. prosthesis involved or only the surrounding area), and uptake pattern (i.e. heterogeneous, diffuse, linear, homogenous, focal, and/or patchy) [7–9, 12].

**Table 1:** [^18^F]FDG-PET/LDCT report characteristics

Criteria
Diagnostic imaging comparison (*with other imaging modalities if available*)
Locoregional involvement *(lymph nodes, abscess, soft tissue)*
Area of interest
Uptake intensity *(uptake similar to background, low, moderate, strong)*
Organ involvement *(i.e. enteric fistula)*
Demarcation *(which vessels affected, what side *etc*.)*
Physiologic distribution
Prosthesis involvement
Body compartments *(head/neck, thorax, abdomen/pelvis, musculoskeletal)*
Uptake pattern *(heterogeneous, diffuse, linear, homogenous, focal, patchy)*

All original reports (written by nuclear physicians) were assessed and scored on assigned criteria by the first two authors (SL and DL). Consecutively, the scored reports were re-checked by an experienced nuclear medicine physician (RS) in case of uncertainties. The predefined (both general and specific) criteria were scored in three categories, consisting of 1. equivocal: the criterion was evaluated by the nuclear medicine physician, but not interpreted (i.e. when images assessed using the specific criterion were not clearly suggestive or not suggestive for VGEI); 2. evaluated: the criterion was evaluated and interpreted (i.e. when the application of the specific criterion allowed proper scoring of VGEI or normal findings); 3. non-evaluated: the criterion was not evaluated (i.e. when the specific criterion was not used for the imaging interpretation).

### [^18^F]FDG-PET/LDCT-conclusions and diagnosis

In order to investigate the level of agreement between the [^18^F]FDG-PET/LDCT conclusions and diagnosis, the conclusions of the reports were scored according to three categories: 0 if the imaging was equivocal (nuclear physician not being able to diagnose or rule out VGEI) for VGEI; 1, meaning the nuclear physician concluded that the [^18^F]FDG-PET/LDCT scan was positive for a VGEI; and 2, meaning the nuclear physician concluded that the [^18^F]FDG-PET/LDCT scan was negative for a VGEI. The final diagnosis of VGEI was proven or rejected according to the MAGIC criteria (VGEI was proven if there was at least one single major criterion and any other criterion from another category) [13]. Each MAGIC category (i.e. clinical and surgical, radiology, and laboratory) was scored (major, minor, or negative). Sensitivity, specificity, and accuracy were calculated for the total group and for subgroups (i.e. equivocal, positive, or negative conclusion), including completely evaluated reports (10 criteria evaluated) and less evaluated reports (≤ 8 criteria evaluated). These cut-off points were chosen to compare two groups with a sufficient number of reports and optimal separation between high and low scores (exclusion of scans with a score of 9 criteria).

### Statistical analysis

Normal distributed continuous variables were reported as mean ± standard deviation and variables with a skewed distribution were reported as median and interquartile range (written as 25th percentile–75th percentile). The distribution of continuous variables was checked visually using histograms and supplemented by the Shapiro–Wilk test. Categorical variables were presented as numbers with accompanying percentages. To compare the conclusion of the [^18^F]FDG-PET/LDCT with the diagnosis of VGEI based on the MAGIC criteria, Cohen’s kappa (non-weighted), sensitivity, and specificity were calculated. Levels of agreement for Cohen’s kappa were < 0, poor; 0.01–0.20, slight; 0.21–0.40, fair; 0.41–0.60 moderate; 0.61–0.80, substantial; and 0.81–1.00, almost perfect. Statistical significance was set at *alpha* < 0.05. Statistical analyses were performed using SPSS (IBM Corp. Released 2022. IBM SPSS Statistics for Windows, Version 29.0. Armonk, NY: IBM Corp).

## Results

### Patient characteristics

In total, 152 patients with the suspicion of VGEI underwent a [^18^F]FDG-PET/LDCT scan and were included in this study. The mean age of the total group was 68.6 ± 8.8 years and 84.9% were male. Fifty-eight (38.2%) patients were current smokers, 84 (55.3%) patients had hypertension, 56 (36.8%) had hyperlipidaemia, and 39 (25.7%) had diabetes mellitus (Table 2).

**Table 2:** Patient characteristics

Patient characteristics	*N* (%) or mean ± SD
Number of patients	152
Age in years	68.6 ± 8.8
Sex (males)	129 (84.9)
BMI in kg/m^2^	26.0 ± 4.7
Tobacco use	58 (38.2)
Hypertension	84 (55.3)
Hyperlipidaemia	56 (36.8)
Diabetes mellitus	39 (25.7)

*N*, number; *SD*, standard deviation; *BMI*, body mass index; *kg*, kilogram; *m*, meter

### Index surgical procedure and [18F]FDG-PET/CT-reporting

Sixty-five percent (*n* = 99) of the patients received a central graft at the index procedure and the remaining patients a peripheral graft (*n* = 53, 34.9%). Seventy-two percent (*n* = 109) underwent open surgical repair (*n* = 104 synthetic prostheses, *n* = 2 bovine pericardial prostheses, *n* = 2 Omniflow® II biosynthetic grafts, and *n* = 1 autologous venous reconstruction) and 28.3% (*n* = 43) endovascular (all synthetic endografts). The median interval from index surgery until [^18^F]FDG-PET/LDCT scan was 35.5 (7.3–73.3) months, with 82.2% (*n* = 125) of the scans defined as late. Forty percent (*n* = 60) of the patients received antibiotic therapy at the time of the [^18^F]FDG-PET/LDCT scan. Over the whole study period, in total 12 different nuclear medicine physicians were involved in the reporting. The nuclear medicine physicians had median 8 (3–15) years of experience at time of reporting the scans and analysed with a median of 12.5 (1.3–19.3) [^18^F]FDG-PET/LDCT scans in patients with a suspicion of VGEI.

### Evaluation of [^18^F]FDG PET/CT-criteria

Fourteen [^18^F]FDG-PET/LDCT reports from external hospitals were not available for scoring and were excluded from this part of the analysis (only the conclusion was present). In Table 3 an overview of the pre-selected criteria is shown. In total, 63 (45.7%) [^18^F]FDG-PET/LDCT reports had 100% score, meaning that all pre-selected criteria were evaluated. Diagnostic imaging comparison was the most often evaluated criterion in 100% of the reports. This criterion was followed by locoregional involvement (99.3% evaluated), region of interest (98.6% evaluated), and uptake intensity (97.8% evaluated). FDG-uptake pattern was the least evaluated criterion (59.4%).

**Table 3:** Evaluated [^18^F]FDG-PET/LDCT criteria

Characteristics (*N* = 138)^a^	Equivocal *N* (%)	Evaluated *N* (%)	Non-evaluated *N* (%)
Diagnostic imaging comparison	0 (0)	138 (100)	0 (0)
Locoregional involvement	0 (0)	137 (99.3)	1 (0.7)
Area of interest	0 (0)	136 (98.6)	2 (1.4)
Uptake intensity	0 (0)	135 (97.8)	3 (2.2)
Organ involvement	0 (0)	134 (97.1)	4 (2.9)
Demarcation	1 (0.7)	130 (94.2)	7 (5.1)
Physiologic distribution	0 (0)	129 (93.5)	9 (6.5)
Prosthesis involvement	14 (10.1)	122 (88.4)	2 (1.4)
Body compartments	0 (0)	121 (87.7)	17 (12.3)
FDG uptake pattern	0 (0)	82 (59.4)	56 (40.6)

^a^Fourteen scans were from external hospitals; therefore, the report was missing

**Fig. 1:**
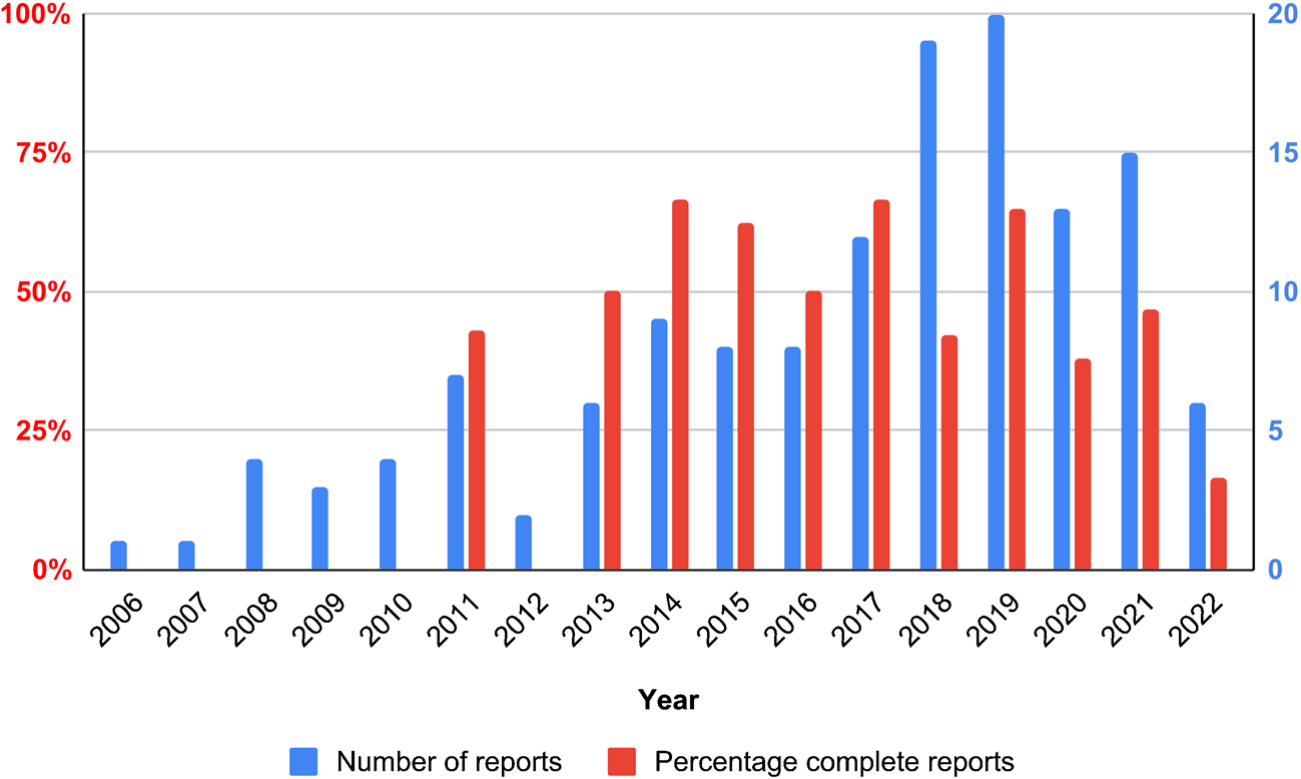
Number of [^18^F]FDG-PET/LDCT reports for suspected vascular graft and endograft infection and percentage of reports in which all criteria were evaluated over the years

A positive trend over the years was observed in number of [^18^F]FDG-PET/LDCT reports for suspected VGEI, see Fig. 1. Furthermore, the percentage of reports in which all criteria were evaluated is shown, and fluctuated per year (Fig. 1). The highest percentages of complete reports (66.7%) were observed in 2014 and 2017.

### Definitive diagnosis of VGEI

In total, 123 (80.9%) patients were diagnosed with VGEI according to the MAGIC criteria. The clinical and surgical MAGIC category scored major in 70 (56.9%) patients, minor in 13 (10.6%) patients, and negative in 40 (32.5%) patients. In the radiology MAGIC category, 55 (44.7%) patients scored major, 60 (48.8%) scored minor, and 8 (6.5%) scored negative. In the laboratory category, 83 (66.7%) scored major and 41 (33.3%) scored minor. Eighty-nine (81.7%) patients with an open graft got the diagnosis VGEI and 34 (79.1%) patients with an endovascular graft (*p* = 0.819). In patients with a diagnosed VGEI, the most common VGEI-specific characteristic was soft-tissue induration (*n* = 46, 37.4%), followed by 22 (17.9%) patients with an abscess, 18 (14.6%) patients with positive FDG-uptake in peri-prosthetic lymph nodes, and 17 (13.8%) patients with a fistula. The [^18^F]FDG-PET/LDCT conclusions and definitive diagnosis (based on the MAGIC criteria) are shown in Table 4. This resulted in a Cohen’s kappa of 0.64 (moderate agreement). In total, 9 (5.9%) reports had an equivocal conclusion, 116 (76.3%) reports had a positive conclusion (i.e. suspected VGEI), and 27 (17.8%) reports a negative conclusion (i.e. not suspected VGEI). When adding the equivocal [^18^F]FDG-PET/LDCT conclusions to the false positives or false negatives, the general performance of [^18^F]FDG-PET/LDCT for the detection of VGEI resulted in a sensitivity of 91%, a specificity of 72%, and an accuracy of 88%. In the false-negative group, 5 (83.3%) patients were on antibiotic therapy at time of the [^18^F]FDG-PET/LDCT scan. When evaluating the diagnostic value of [^18^F]FDG-PET/LDCT reports with lowest number of criteria evaluated (≤ 8 evaluated criteria, *n* = 26) and reports with the highest number of criteria evaluated (all criteria evaluated, *n* = 63), a sensitivity of 83%, a specificity of 50%, and an accuracy of 73% were observed for the less-evaluated reports and a sensitivity of 92%, a specificity of 77%, and an accuracy of 89% were observed for the completely evaluated reports. In the completely evaluated group, there were no equivocal [^18^F]FDG-PET/LDCT conclusions noted, and in the less evaluated group five equivocal conclusions were present.

**Table 4:**
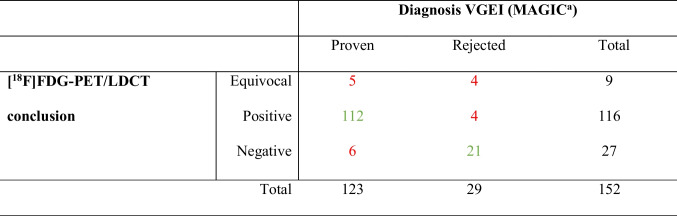
[^18^F]FDG-PET/LDCT conclusions and definite diagnosis according to the MAGIC criteria*

*[*^*18*^*F]FDG-PET/CT* 2-deoxy-2-[^18^F]fluoro-D-glucose-positron emission tomography and low dose computed tomography, *VGEI* vascular graft and endograft infection, *MAGIC* Management of Aortic Graft Infection

^a^At least one single major criterion and at least one minor criterion from another category. Sensitivity: 91%, specificity: 72%

### VGEI treatment

Eighty-six (69.9%) of the 123 patients with a diagnosed (according to the MAGIC criteria) VGEI underwent surgical treatment in addition to antibiotic therapy. From the (diagnosed VGEI) patients who underwent surgical treatment, 80 (93.0%) patients got intraoperative tissue or graft cultures of which 66 (82.5%) were positive. In 41 (62.2%) patients, the culture result was polymicrobial, in 23 (34.8%) patients it was monomicrobial, and in two (3.0%) patients the culture results were positive, but the microorganism was missing. The other patients were treated with antibiotics alone. The latter group of patients were often not fit enough to underwent surgical repair or were clinically stable with antibiotic suppression therapy. The median interval from [^18^F]FDG-PET/LDCT scan to surgical VGEI treatment was 27.5 (7.0–90.5) days.

## Discussion

In this retrospective study, we assessed the completeness of [^18^F]FDG-PET/LDCT reports of suspected VGEI patients based on ten predefined criteria as reported in current literature. Less than half of all [^18^F]FDG-PET/CT reports contained all criteria. The least frequently assessed criterion was the pattern of [^18^F]FDG uptake, despite its critical significance to determine the diagnosis of VGEI [12]. A sensitivity of 91% and specificity of 72% were found in the overall cohort, which is comparable with the existing literature [3, 5, 7, 16]. Furthermore, a higher sensitivity and specificity in fully evaluated reports compared to reports using fewer evaluation criteria were observed. This is an important finding since it demonstrates the additional value of striving for standardised reporting including all predefined criteria to increase the diagnostic accuracy. Accordingly, standardised and complete reports should be recommended in the new guidelines for patients with suspected VGEI.

The importance of the uptake pattern has been already addressed when [^18^F]FDG-PET/LDCT or [^18^F]FDG-PET/CTA are used in other clinical situations, such as in the diagnosis of infectious endocarditis and in patients with suspected infections after a Bentall procedure [17, 18]. In these settings careful assessment of the presence of persistent host versus biomaterial coating reaction, the sewing ring of the valve, chronic tension, or friction exerted on anchor points as well as of all the factors affecting the intensity of [^18^F]FDG uptake (i.e. time elapse from surgery, surgical and post-surgical complications, ongoing antimicrobial treatment, specific strains) has been demonstrated of fundamental to maintain high specificity when using [^18^F]FDG [19]. If the proper protocol for patients’ preparation and imaging acquisition are followed and specific imaging interpretation criteria are used, sensitivity and specificity can reach 91% in case of infective endocarditis and 97% and 73% in case of Bentall procedures [17, 18].

The presence of para-physiologic, [^18^F]FDG uptake along the wall of the vascular grafts representing reactive granulomatosis is often visible and validated semi-quantitative SUV cut-off points are lacking. Therefore, describing the uptake pattern remains of utmost importance [7, 16, 20]. The uptake pattern has a comparable sensitivity, but a significant higher specificity compared to the common description of the intensity of [^18^F]FDG-uptake against the SUVmax or tissue-to-background ratio (TBR) [16, 21]. Indeed, focal or heterogeneous uptake along the vessel is a hallmark of VGEI as compared to linear, diffuse, and homogenous uptake which does in general not represent infection [11, 22, 23]. Therefore, to increase the diagnostic accuracy of [^18^F]FDG-PET/LDCT in VGEI, it is necessary to provide a combination of visual uptake pattern with [^18^F]FDG-uptake intensity [7]. The some less frequently observed criterion of [^18^F]FDG-uptake pattern in the current study is maybe due to dated reports with less attention to uptake patterns in VGEI. The [^18^F]FDG-uptake intensity in our cohort was scored using a four-point scale which has been validated in our centre, used for several years, and has been recommended in previously published literature [7]. Recently, in 2015, Sah et al. proposed a new scoring method that consists of a five-point scale [24]. In the future, researchers in the field of [^18^F]FDG-PET should be aware of this and a comparison should be made between these two scoring methods.

A recently published study has shown that the presence of positive (defined as follows: visual uptake of grade two or four and/or a short axis diameter > 10 mm on LDCT) locoregional lymph nodes on [^18^F]FDG-PET/CT imaging has a high specificity (96%) and positive predictive value (95%) for VGEI [25]. However, these findings were accompanied by a low sensitivity. Therefore, the positive locoregional lymph nodes could have a positive influence on the specificity of new interpretation criteria. The current study corroborates to the conclusion that further research is needed to evaluate the diagnostic accuracy of lymph nodes for detecting and diagnosing VGEI, as positive lymph nodes in the area surrounding the vascular graft were observed in only 14.6% of the patients with a diagnosed VGEI.

In the first half of the study period, an increase in both numbers of reports and percentages of completely evaluated reports was noted. The decrease that was observed from 2020 was probably due to the COVID-19 pandemic which resulted in lower patient admissions. The increase of number of reports might be due to the fact that over time there was more knowledge about the value of [^18^F]FDG-PET/LDCT in the diagnosis of infection. In 2011, the first report that met all criteria was observed. In this year, the Department of Nuclear Medicine at the UMCG implemented a systematic method of reporting according to body compartments. The increased percentage of completely evaluated reports over the years could be caused by developments in imaging modalities and/or due to the introduction of multidisciplinary consultation (including a vascular surgeon, a microbiologist, an infectiologist, a radiologist, and a nuclear medicine physician) of VGEI patients. Another explanation could be an increased knowledge on patterns that can be observed on [^18^F]FDG-PET/LDCT. Despite the fact that there is a slight upward trend in reporting VGEI, < 50% of the reports contained all criteria, while the report is often the only way of communication between the nuclear medicine physician and the clinician (in this case, the vascular surgeon) [26]. This indicates that there is a strong need to improve the reporting, including a more systematic reporting approach with standardised interpretation criteria. As described earlier by the European Association of Nuclear Medicine and the European Association of Cardiovascular imaging, it is crucial that the referring clinician understands the report as intended by the nuclear medicine physician, as this approach is already more common in conventional nuclear cardiology [27], and in PET/CT applications in oncology [8–10, 28]. Reporting of several [^18^F]FDG-PET/CT applications in cardiovascular diseases are less well addressed, as in the current situation with VGEI, but also in other infections and inflammatory diseases, such as (infective) endocarditis, infection of cardiac implantable electronic devices, large vessel vasculitis, and polymyalgia rheumatica [29, 30]. Although interpretation for several inflammatory-, infective-, infiltrative-, and device-related diseases is described, specific and user-friendly recommendations on reporting are often incomplete [31]. As highlighted now for VGEI, recommendations on reporting with standardised interpretation criteria should be compiled for these missing parts of [^18^F]FDG-PET/CT applications in cardiovascular diseases. An example of user-friendly, standardised reporting standards for nuclear imaging on cardiac amyloidosis are published by Dorbala et al. [32, 33]. It is recommended to write the report clearly and as simple as possible, with a limited number of abbreviations, with quantified data instead of qualitative (e.g. small, large, slightly) descriptions (if possible), and with less as possible defensive expressions (e.g. cannot be excluded) [27].

One hundred twenty-three (80.9%) out of 152 patients were diagnosed with VGEI. This high proportion is due to the fact that CTA is still the gold standard imaging modality in suspected VGEI [3]. An [^18^F]FDG-PET/LDCT scan was performed subsequently to confirm the diagnosis and/or to evaluate the extent of the infection. As a consequence, there is a selection bias with a high prevalence of VGEI in this suspected VGEI cohort. Almost three-quarter of the patients in our cohort got a graft infection after an open surgical procedure, most likely explained by an overall higher incidence after open surgical repair compared to endovascular repair. For open aortic repair the incidence is up to 4.5% versus 0.3–1.0% for endovascular aortic repair [34]. One of the reasons for this difference is the large, longer lasting surgical wound in open procedures.

**Fig. 2:**
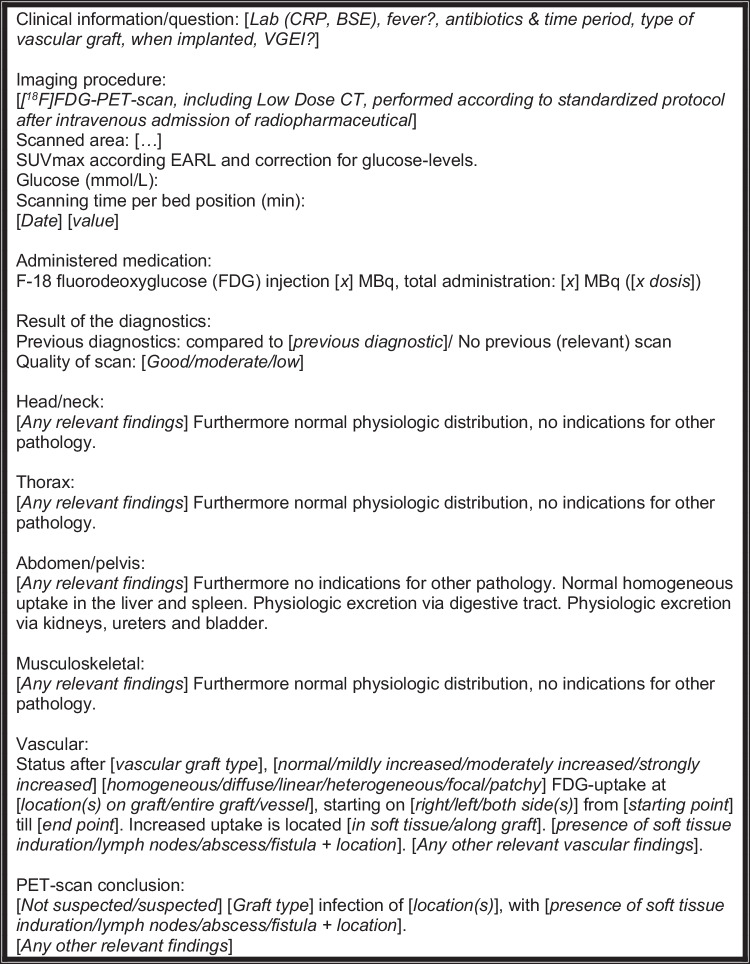
Recommendation for a concise and complete [^18^F]FDG-PET/LDCT report for vascular graft and endograft infection

Based on the results of the current study and according to available literature (VGEI specific and [^18^F]FDG-PET/CT broad), we provide a first recommendation for a concise and complete [^18^F]FDG-PET/LDCT report for VGEI (Fig. 2) [7–9, 12, 31–33]. Standardisation of nuclear medicine reporting in the wide field of cardiovascular diseases should follow as well and this proposed reporting standard can serve as format for new reporting standards on other cardiovascular diseases, such as infective native aortic aneurysm, where there is a potential role of and value in performing a [^18^F]FDG-PET/LDCT, but a lack of studies in the field [35].

### Limitations

This study has some limitations. The first limitation is the retrospective nature, which causes a lower level of evidence compared to other study designs. However, the original [^18^F]FDG-PET/LDCT reports had been used, which were prospectively analysed by the nuclear medicine physician. Furthermore, a limitation of this study is the use of the MAGIC criteria, since these criteria are originally validated for aortic graft infection instead of peripheral graft infection [13]. The MAGIC criteria were later found to be useful as well for peripheral grafts [36]. However, the specificity was lower compared to central grafts [36]. Another limitation is the heterogeneity of the patients with different grades of infection, types of surgery, graft locations, and graft materials. Another limitation is that 27 (17.8%) patients had an early [^18^F]FDG-PET/LDCT scan (< 4 months), while a previously published study has shown that scans in the early postoperative phase may have a high false positive rate [7]. More than half of the patients used antibiotics during the time of their [^18^F]FDG-PET/LDCT scan and in the false-negative group even 83.3% used antibiotics during the scan. This may have resulted in an underestimation of the prevalence of VGEI specific characteristics, because antibiotic therapy can induce a decrease in metabolic activity of the infection [24]. This decrease might have increased the number of false negative reports, which can lead to undertreatment of VGEI patients.

## Conclusions

In this study, < 50% of the [^18^F]FDG-PET/LDCT reports of patients with a suspected VGEI met the predefined criteria for being complete. This led to a lower sensitivity and specificity in comparison with complete reports. Implementing a specific recommendation for VGEI reporting is therefore needed in a next VGEI guideline update [7]. Based on the results of the current study and accompanying literature, we provided a first recommendation for a concise and complete [^18^F]FDG-PET/LDCT report for VGEI. Standardisation of [^18^F]FDG-PET/LDCT reporting is warranted to improve accuracy and to reduce heterogeneity between different medical centres and to allow comparison between studies.

## Data Availability

The datasets generated during and/or analysed during the current study are available from the corresponding author on reasonable request.
